# Integrated Bacterial and Fungal Diversity Analysis Reveals the Gut Microbial Alterations in Diarrheic Giraffes

**DOI:** 10.3389/fmicb.2021.712092

**Published:** 2021-08-12

**Authors:** Aoyun Li, Bingxian Liu, Feiran Li, Yuanyuan He, Lei Wang, Muhammad Fakhar-e-Alam Kulyar, Huade Li, Yuhang Fu, Huaisen Zhu, Yaping Wang, Xiong Jiang

**Affiliations:** ^1^Hubei Three Gorges Polytechnic, Yichang, China; ^2^College of Veterinary Medicine, Huazhong Agricultural University, Wuhan, China; ^3^College of Veterinary Medicine, South China Agricultural University, Guangzhou, China; ^4^Animal Husbandry Station of Bijie City, Bijie, China; ^5^Sichuan Academy of Grassland Science, Chengdu, China

**Keywords:** gut microbiota, diarrhea, 16S rDNA, ITS, giraffe

## Abstract

Gut microbiota has been demonstrated to be associated with multiple gastrointestinal diseases, but information regarding the gut microbial alternations in diarrheic giraffe remains scarce. Here, 16S rDNA and ITS gene amplicon sequencing were conducted to investigate the gut microbial composition and variability in diarrheic giraffes. Results demonstrated that *Firmicutes* and *Proteobacteria* were the most dominant phyla in the gut bacterial community, whereas *Ascomycota* and *Basidiomycota* were observed to be predominant in the gut fungal community regardless of health status. However, the species and relative abundance of preponderant bacterial and fungal genera in healthy and diarrheic giraffes were different. In contrast to the relatively stabilized gut fungal community, gut bacterial community displayed a significant decrease in the alpha diversity, accompanied by distinct changes in taxonomic compositions. Bacterial taxonomic analysis revealed that the relative abundances of eight phyla and 12 genera obviously increased, whereas the relative abundances of two phyla and eight genera dramatically decreased during diarrhea. Moreover, the relative richness of five fungal genera significantly increased, whereas the relative richness of seven fungal genera significantly declined in diarrheic giraffes. Taken together, this study demonstrated that diarrhea could cause significant alternations in the gut microbial composition of giraffes, and the changes in the gut bacterial community were more significant than those in the gut fungal community. Additionally, investigating the gut microbial characteristics of giraffes in different health states is beneficial to provide a theoretical basis for establishing a prevention and treatment system for diarrhea from the gut microbial perspective.

## Introduction

Ruminant intestines are colonized by trillions of microbes which play crucial roles in immune system maturation, intestinal epithelial mucosal barrier maintenance, metabolism, and nutrient absorption (Garrett et al., 2010; Arumugam et al., 2011; Tremaroli and Backhed, 2012; Backhed et al., 2015). Statistical analysis indicates that approximately 98% of intestinal microbes are bacteria, whereas the remaining contains fungi (about 0.1%), protozoa, and viruses (Garrett et al., 2010; Tremaroli and Backhed, 2012; Backhed et al., 2015). These microbes display a symbiotic relationship with the host through complicated networks of interactions and crosstalk with each other (Aziz et al., 2013; Guo et al., 2015). Remarkably, some potentially pathogenic microorganisms may also inhabit as parts of normal gut microbiota but may take opportunity to cause disease, e.g., gut microbial alteration and immune dysregulation of host (Aziz et al., 2013; Guo et al., 2015). Gut microbial community evolves with host and has become a vital organ affecting its health (Cryan and Dinan, 2012; Zhang et al., 2020). Several studies provided supporting evidence that shifts in the gut microbial composition that could extend its adverse effects beyond the gastrointestinal system and affect the functions of extra-intestinal organs, such as the brain and liver (Zhang et al., 2020). Gut microbial community has been demonstrated to be involved in the development of multiple diseases such as colorectal cancer, diabetes, obesity, and dyspepsia (Delzenne et al., 2011; Musso et al., 2011; Ambalam et al., 2016). Although gut bacterial importance has been well demonstrated by numerous studies, analyses regarding the relationship between gut fungal communities and host health have been insufficient to date.

Diarrhea is one of the leading causes of decreased productivity and death in ruminants that has been considered a vital factor impeding animal husbandry development in many countries (Musso et al., 2011; Ambalam et al., 2016). Early investigations revealed that diarrhea was present in almost all ruminants and especially epidemic in neonatal goat, sheep, cattle, and yak with immature gastrointestinal tract, which caused approximately half of all ruminant deaths (Li et al., 2018; Bu et al., 2020; Xue et al., 2020). Previous research has indicated that some intestinal microbes including bacteria and fungi of ruminants alternate between preponderant and weak populations accompanied by diarrheic symptoms (Yang et al., 2017; Wang et al., 2018; Wang et al., 2019a). Therefore, some inevitable associations may be present between alternations in gut microbial community and diarrhea, but its specific connections and laws remain to be determined. Previously, numerous studies were performed on pathogenic bacteria, parasite, and virus to reveal the cause of ruminant diarrhea (Gallardo et al., 2020; He et al., 2020). Recent studies on gut microbiota of goat, yak, and mice have provided evidence that gut microbial dysbiosis may be one of the reasons of diarrhea (Han et al., 2017; Shao et al., 2020).

Metagenomic analysis based on high-throughput sequencing technology is an efficient tool for characterizing gut microbial composition and diversity differences after suffering certain diseases and has made possible in systematically investigating the relationship between host diseases and gut microbiota (Han et al., 2017; Shao et al., 2020). Moreover, in-depth comparison and analysis of obtained gut microbial information contributed to further understand the mechanisms causing ill health and develop the strategies to minimize the collateral damage (Han et al., 2017; Shao et al., 2020). Giraffe (*Giraffa camelopardalis*) mainly inhabiting the African continent is the tallest terrestrial ruminant in the world and displays a complicated gastrointestinal microbial ecosystem (Brown et al., 2007; AlZahal et al., 2016). This species has been introduced into zoos around the world but showed a high diarrheic rate due to the alterations in habitat and survival environment (Brown et al., 2007; Mulherin et al., 2008; AlZahal et al., 2016). However, to date, the relationship between gut microbial composition and diversity and diarrhea in giraffes is still unclear. Taking advantage of this gap, we investigated the composition and variability of gut bacterial and fungal communities in the healthy and diarrheal giraffes by 16S rDNA and ITS2 amplicon sequencing.

## Materials and Methods

### Sample Acquisition

In this study, a total of twelve 4-year-old giraffes inhabiting in Yichang zoo (Yichang, China) were used for sample collection, including six healthy giraffes and six diarrheic populations. Additionally, the proportion of male and female in both groups was 1:1. These giraffes were maintained under the same conditions and possessed the same immune procedure. Prior to the sample acquisition, all the giraffes were observed and diagnosed by professional veterinarians to determine their health status and diarrheic populations without any medicinal treatment. Sufficient feed and water were provided *ad libitum* for all giraffes throughout the experimental period. One day prior to sample acquisition, healthy and diarrheic giraffes were placed in separate pens to prevent infection and sample contamination. Six individual fresh fecal samples were collected from each giraffe *via* using sampler the following morning. Subsequently, the obtained rectal feces were resampled from the intermediate portion to minimize pollution through bedding and flooring. Finally, all the samples were placed immediately in sterile plastic containers, snap-frozen using liquid nitrogen and stored at −80°C for subsequent DNA extraction.

### 16S rDNA and ITS Gene Amplification and Sequencing

The bacterial and fungal genomic DNA was extracted from feces of giraffes using QIAamp DNA Mini Kit (QIAGEN, Hilden, Germany) following suggested instructions of manufacturer. The total microbial DNA was quantified *via* utilizing UV-Vis spectrophotometer (NanoDrop 2000, United States), and DNA integrity was evaluated by 0.8% agarose gel electrophoresis. Bacterial 16S rDNA (338F: ACTCCTACGGGAGGCAGCA and 806R: GGACTACHVGGGTWTCTAAT) and fungal ITS gene primers (ITS5F: GGAAG TAAAAGTCGTAACAAGG and ITS2R: GCTGCGTTCTTCATCGA TGC) with special barcodes were synthesized on the basis of the conservative regions to amplify the V3/V4 and ITS2 regions, respectively. To ensure the accuracy of the results, PCR amplification was conducted in triplicates under the same conditions. The agarose gel electrophoresis (2%) and AxyPrep DNA Gel Extraction Kit (Axygen, Union City, CA, United States) was employed to evaluate PCR amplification product and recycle target fragment, respectively. PCR-recycled products were performed fluorescent quantitation on Microplate reader (BioTek, FLx800) based on the original electrophoretic results. According to the fluorescence quantitative results and the requirements of sequencing quantity, the samples were mixed in corresponding proportions. The obtained PCR products were used to construct the sequencing library *via* using TruSeq Nano DNA LT Library Prep Kit (Illumina, San Diego, CA, United States). Meanwhile, the 2% agarose gel electrophoresis was used for the final fragment selection and purifying the library. The quality inspection and fluorescence quantification of the sequencing libraries were performed before sequencing. The qualified libraries were diluted and then mixed in corresponding proportions based on the required sequencing quantity. The final libraries were subjected to high-throughput sequencing *via* MiSeq sequencing machine.

### Bioinformatics and Statistical Analysis

The original data produced from high-throughput sequencing were performed by quality screening using QIIME software (Qiime1.9.1) to achieve reliable results in the subsequent bioinformatics analysis. The interrogative sequences including short sequences (<200 bp), mismatched primers, and chimera were discarded utilizing QIIME software (Qiime1.9.1), and the qualified sequences were assigned to the corresponding individuals according to the information of primer and barcode. The obtained sequences were OTU partitioned and clustered based on 97% similarity, and the representative sequences were conducted through classification identification and phylogenetic analysis. The gut microbial alpha diversity was calculated according to the relative abundance distribution of OTU in each sample. Beta diversity was calculated based on weighted and unweighted UniFrac distance to evaluate the difference and similarity in different samples and groups. Simultaneously, the rarefaction curve of each sample was generated to assess the sequencing depth. Statistical analysis of data was performed using R (v3.0.3) and GraphPad Prism (version 7.0c). *p*-values < 0.05 were considered statistically significant, and the values were presented as means ± SD.

## Results

### Data Acquisition and Analysis

**FIGURE 1:**
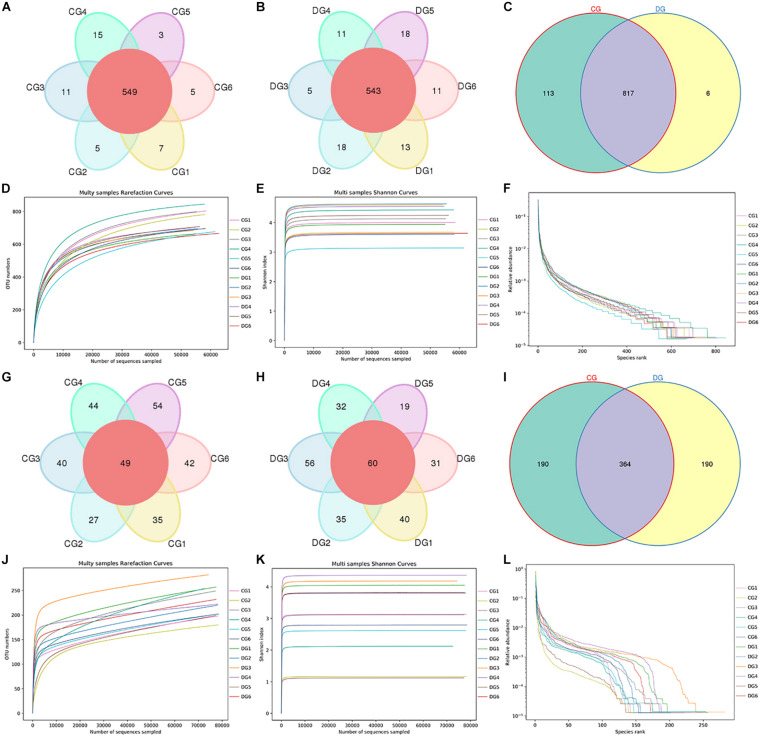
Gut bacterial and fungal OTU distribution and feasibility analysis. **(A–C)** Venn diagrams for gut bacterial OTU distribution. **(D–F)** Bacterial rarefaction and rank abundance curves were used for assessing the quality of sequencing including depth, abundance, and evenness. **(G–I)** Venn diagrams for gut fungal OTU distribution. **(J–L)** Fungal rarefaction and rank abundance curves were used to evaluate the quality of sequencing including depth, abundance, and evenness. Each colored curve displayed in the figures represents a sample.

In the present microbiome investigation, 12 fecal samples from healthy and diarrheic giraffes were subjected to amplicon sequencing and a total of 958,862 (CG = 479,660, DG = 479,202) and 960,350 (CG = 480,389, DG = 479,961) original sequences were acquired from the V3/4 and ITS2 regions, respectively (Table 1). After sequence filtering, 1,795,319 high-quality reads were totally acquired from all the samples, with an average of 70,620 (ranging from 68,577 to 72,501) and 78,989 (varying from 78,434 to 79,841) reads from bacterial and fungal populations per sample, respectively (Table 2). Following taxonomic assignment, a total of 936 bacterial OTUs and 744 fungal OTUs were recognized based on 97% nucleotide-sequence similarity (Figures 1A,B,G,H). Moreover, we also observed 817 and 364 core OTUs in the bacterial and fungal communities, which accounted for approximately 87.29% and 48.92% of the total OTUs, respectively (Figures 1C,I). Both species accumulation and rarefaction curves in per sample were relatively flat and showed a tendency to saturate characteristics, indicating that nearly all the bacterial and fungal species were identified in fecal samples of giraffes (Figures 1D,E,J,K). Furthermore, the rank abundance curves for all samples were wide and fell gently, suggesting satisfactory evenness and abundance (Figures 1F,L).

**TABLE 1:** The bacterial sequence information of each sample.

**Sample**	**Raw reads**	**Clean reads**	**Effective reads**	**AvgLen (bp)**	**GC (%)**	**Effective (%)**
CG1	79,898	79,389	70,069	422	53.25	87.7
CG2	79,747	79,277	68,993	422	53.65	86.51
CG3	79,912	79,401	68,577	420	53.05	85.82
CG4	80,066	79,565	70,631	418	53.71	88.22
CG5	79,912	79,286	70,064	424	53.28	87.68
CG6	80,125	79,660	69,945	420	52.79	87.29
DG1	79,811	79,378	69,500	421	54.07	87.08
DG2	79,677	79,196	71,013	419	54.38	89.13
DG3	79,983	79,528	72,110	418	54.42	90.16
DG4	79,699	79,288	72,501	419	54.2	90.97
DG5	80,049	79,603	72,133	419	54.21	90.11
DG6	79,983	79,497	71,908	421	54.6	89.90

**TABLE 2:** The fungal sequence information of each sample.

**Sample**	**Raw reads**	**Clean reads**	**Effective reads**	**AvgLen (bp)**	**GC (%)**	**Effective (%)**
CG1	80,228	79,749	79,352	228	44.76	98.91
CG2	80,062	79,593	79,557	251	50.72	99.37
CG3	79,851	79,325	79,310	230	44.32	99.32
CG4	79,832	78,434	74,738	255	50.45	93.62
CG5	80,134	79,617	79,494	237	46.7	99.20
CG6	80,282	79,841	79,836	231	46.01	99.44
DG1	80,110	79,630	78,957	237	47.03	98.56
DG2	79,829	79,269	79,242	251	49.41	99.26
DG3	79,679	79,155	79,060	243	47.78	99.22
DG4	80,093	79,569	79,500	246	47.59	99.26
DG5	79,911	79,085	79,057	256	42.15	98.93
DG6	80,339	79,787	79,772	240	48.26	99.29

### Shifts in Gut Microbial Diversities With the Effect of Diarrhea

**FIGURE 2:**
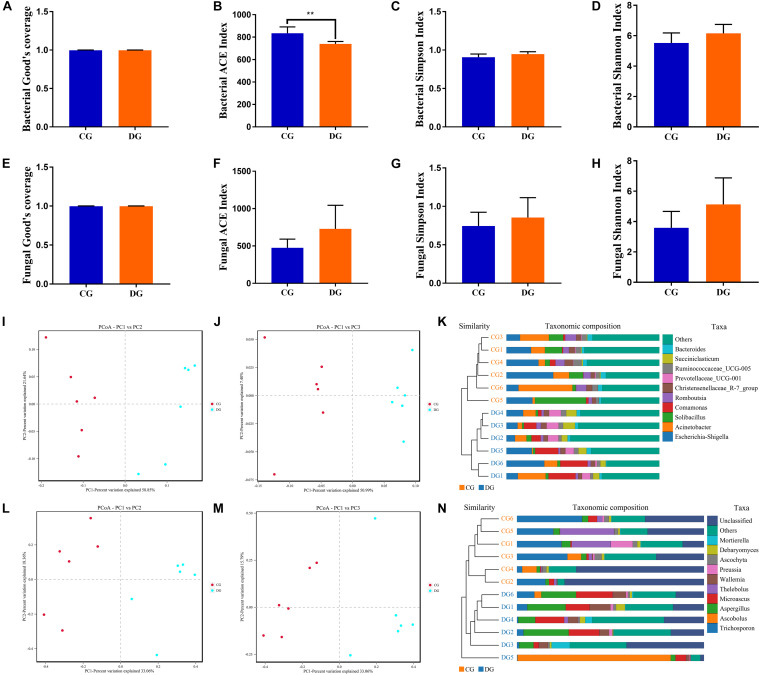
The alternations of gut bacterial and fungal diversities during diarrhea. Gut bacterial and fungal alpha diversities can be evaluated by Good’s coverage **(A,E)**, ACE **(B,F)**, Simpson **(C,G)**, and Shannon **(D,H)**. **(I,J)** Principal coordinate (PCoA) analysis based on the weighted and unweighted UniFrac distance of gut bacterial community. **(L,M)** Principal coordinate (PCoA) analysis of gut fungal community. **(K,N)** Gut bacterial and fungal clustering analysis based on unweighted pair-group method with arithmetic means (UPGMA). All of the data represent means ± SD. **p < 0.01.

To further dissect the alternations of gut bacterial and fungal communities in diarrheic giraffe, the qualified sequences were aligned to estimate alpha and beta diversity indices. Gut bacterial and fungal alpha diversity could be characterized by sequencing depth (Good’s coverage), species abundance (ACE), and species diversity (Shannon and Simpson). Good’s coverage estimates varied from 99.79 to 99.96%, suggesting that the majority of bacterial and fungal phenotypes presented in each sample were detected (Figures 2A,E). There was statistically significant differences in the gut bacterial ACE (834.53 ± 56.67 versus 739.61 ± 21.69, *p* = 0.003) indices, whereas the Simpson index (0.91 ± 0.042 versus 0.95 ± 0.031, *p* = 0.087) and Shannon (5.53 ± 0.65 versus 6.16 ± 0.59, *p* = 0.11) indexes were not dramatically different between the CG and DG groups (Figures 2B–D). Intergroup analysis of alpha diversity intuitively showed that diarrhea observably decreased the gut bacterial abundance but had no effect on the bacterial diversity. Moreover, there was no significant difference in the gut fungal four α-diversity indices in both groups, indicating a relatively stabilized gut fungal community in giraffes during the occurrence of diarrhea (Figures 2F–H). The principal coordinate analysis (PCoA) and unweighted pair-group method with arithmetic means (UPGMA) tree analysis were applied to assess the variabilities and similarity among intergroup and intragroup individuals. Both the weighted and unweighted PCoA plots showed that the individuals in the CG group were clustered closely and separated from the DG group, which was consistent with the results of UPGMA analysis, indicating that the gut bacterial and fungal principal compositions could be strongly influenced by the diarrhea (Figures 2I–N).

### Taxonomic Composition and Alteration of Gut Bacterial Community

**FIGURE 3:**
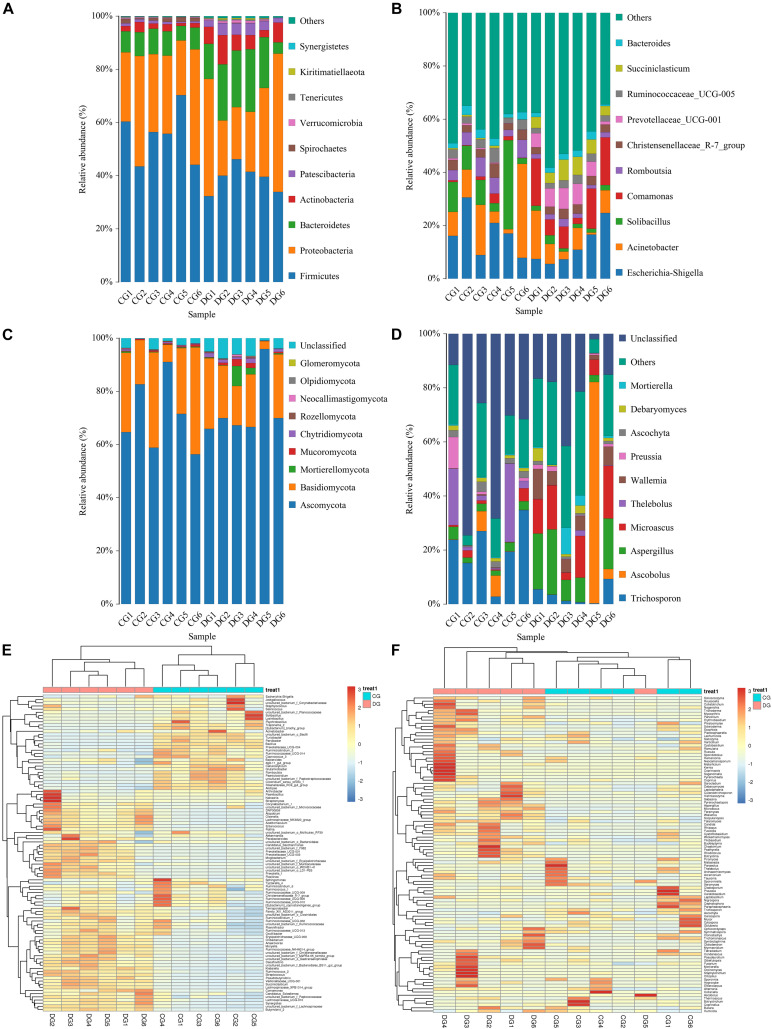
The composition and relative abundance of the gut microbial community at the phylum and genus levels. **(A,B)** The gut bacterial relative abundance and composition of healthy and diarrheic giraffes at the phylum and genus levels. **(C,D)** The gut fungal relative abundance and composition of healthy and diarrheic giraffes at the phylum and genus levels. **(E,F)** Heatmap of the 50 most abundant gut bacterial and fungal genera in healthy and diarrheic giraffes.

The relative proportion of preponderant taxa at the different taxonomical levels were detected through microbial taxon assignment, and significant alterations in the gut bacterial abundances and compositions were observed in both groups. At the phylum level, a total of 17 phyla were identified from the 12 samples, varying from 14 to 17 phyla per sample. Based on the phylum assignment result, *Firmicutes* (55.21%, 38.79%), *Proteobacteria* (31.61%, 32.55%), *Bacteroidetes* (8.19%, 16.79%), and *Actinobacteria* (2.17%, 6.46%) were the four most preponderant phyla in CG and DG groups, which accounted for approximately 95% of the total taxonomic groups identified in all samples (Figure 3A). Other phyla such as *Verrucomicrobia* (0.22%, 0.55%), *Tenericutes* (0.33%, 0.34%), *Kiritimatiellaeota* (0.02%, 0.33%), and *Synergistetes* (0.02%, 0.23%) in both groups were identified in low abundance. To further investigate the effect of diarrhea on taxonomic compositions, 194 genera were detected from the gut microbiota of giraffes. Among these genera identified, *Escherichia-Shigella* (16.98%) was the most predominant bacterial genus in the CG group, followed by *Acinetobacter* (13.11%) and *Solibacillus* (11.63%) (Figure 3B). However, *Escherichia-Shigella* (12.41%), *Acinetobacter* (7.71%), and *Comamonas* (11.36%) were abundantly present in the DG group, accounting for over 31% of overall bacterial composition. The genus-level cluster analysis employing heatmap revealed the distribution of the bacterial genus in different samples and indicated the influence of diarrhea on the bacterial genus-level compositions (Figure 3E).

**FIGURE 4:**
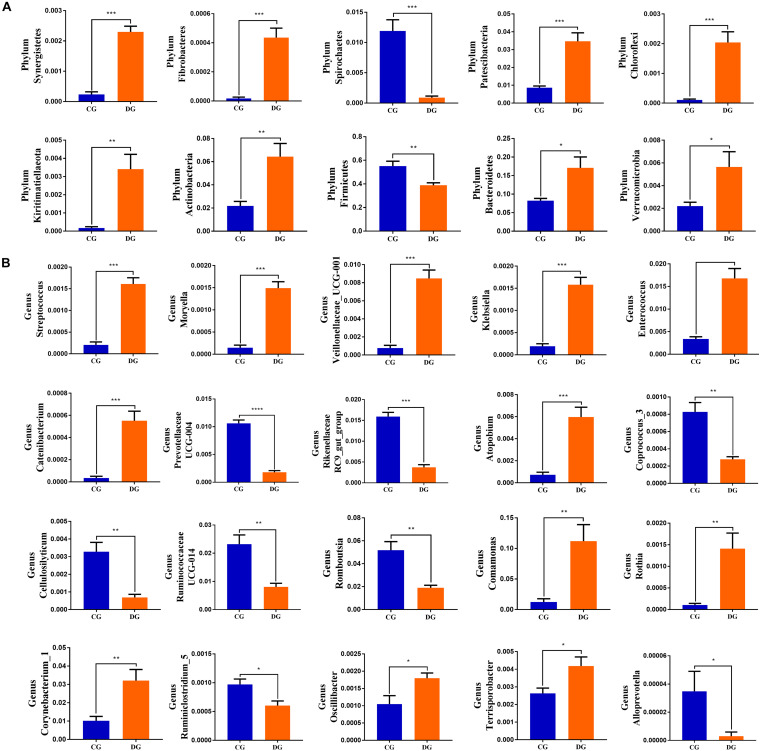
Significant alternations in the gut bacterial abundance at the level of phylum **(A)** and genus **(B)** during diarrhea. All of the data represent means ± SD. *p < 0.05; **p < 0.01; ***p < 0.001 and ****p < 0.0001.

**FIGURE 5:**
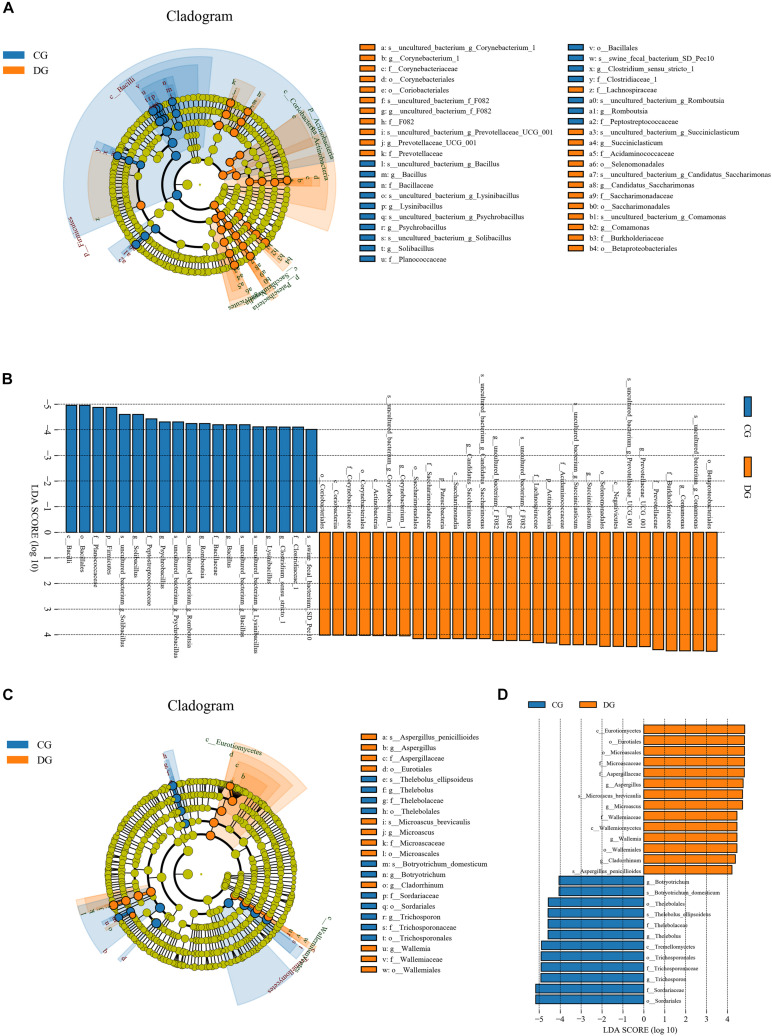
LEfSe analysis integrated with LDA scores revealed differential biomarkers associated with diarrhea in giraffes. **(A,C)** Cladogram revealing the phylogenetic distribution of intestinal bacteria and fungus correlated with the control or diarrheic groups. **(B,D)** The differences in the relative abundance of bacteria and fungi between the control and the diarrheic groups. LDA scores > 4 was considered statistically significant.

To further dissect the shifts in taxonomic compositions of giraffes in different health states, Metastats analysis was performed for different classification levels. A comparison of the DG and CG groups revealed a significant increase (*p* < 0.05 or *p* < 0.01) in the abundance of *Synergistetes*, *Fibrobacteres*, *Patescibacteria*, *Chloroflexi*, *Kiritimatiellaeota*, *Actinobacteria*, *Bacteroidetes*, and *Verrucomicrobia*, as well as a distinct decrease (*p* < 0.01) in the abundance of *Spirochaetes* and *Firmicutes* (Figure 4A). At the genus level, 20 genera were totally identified to be dramatically different between both groups. Of these discriminatory taxa, the relative abundances of 12 bacterial genera significantly increased, whereas the relative abundances of eight bacterial genera dramatically decreased under the influence of diarrhea (Figure 4B). Considering that this discriminant analysis cannot distinguish the dominant taxon, linear discriminant analysis effect size (LEfSe) analysis coupled with linear discriminant analysis (LDA) was conducted to identify the specific bacteria related to diarrhea (Figure 5). Results revealed that at the phylum level, the abundance of *Actinobacteria* and *Patescibacteria* in the DG group were significantly preponderant than the CG group, while the *Firmicutes* was lower. At the genus level, the DG group was significantly enriched for *Comamonas*, *Prevotellaceae_UCG_001*, *Succiniclasticum*, and *Corynebacterium_1*, whereas the CG group showed a significantly higher abundance of *Clostridium_sensu_stricto_1*, *Lysinibacillus*, *Bacillus*, *Romboutsia*, *Psychrobacillus*, and *Solibacillus*.

### Significant Alterations in Gut Fungal Taxonomic Compositions in Diarrheic Giraffe

There were nine phyla and 262 genera identified in the gut fungal communities of all giraffes using RDP classifier. The top 10 phyla and 10 genera of gut fungal community in both groups are presented in Figures 3C,D. The phyla *Ascomycota* (CG = 70.63%, DG = 72.63%) and *Basidiomycota* (CG = 25.84%, DG = 18.01%) were the most dominant fungi in giraffes regardless of health status, which together consisted of over 90% of the fungal composition (Figure 3C). Other fungal phyla such as *Chytridiomycota* (0.28%, 1.07%), *Rozellomycota* (0.16%, 0.25%), *Neocallimastigomycota* (0.11%, 0.18%), *Olpidiomycota* (0.01%, 0.06%), and *Glomeromycota* (0.02%, 0.05%) in the CG and DG groups were represented with a lower abundance. At the genus level, the dominant fungal genera observed in the CG group were *Trichosporon* (20.76%), *Aspergillus* (2.95%), and *Thelebolus* (9.44%), whereas *Ascobolus* (14.33%), *Aspergillus* (13.81%), and *Microascus* (12.14%) were enriched in the DG group (Figure 3D). The heatmap showed a higher similarity of the individuals within group than that among groups and revealed the alternations in fungal genus-level compositions under the effect of diarrhea (Figure 3F).

**FIGURE 6:**
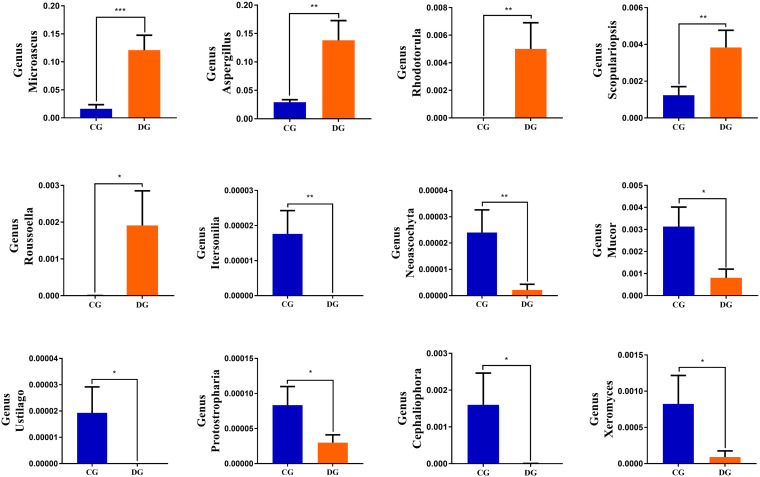
Significant changes in the gut fungal abundance at the level of phylum and genus during diarrhea. *p < 0.05; **p < 0.01 and ***p < 0.001 represent distinct difference between control and diarrheic groups.

Using Metastats analysis to investigate the fungal genus-level taxonomic compositions in both groups, we observed that the relative abundances of five genera (*Microascus*, *Aspergillus*, *Rhodotorula*, *Scopulariopsis*, and *Roussoella*) significantly increased, whereas the relative abundances of seven genera (*Itersonilia*, *Neoascochyta*, *Mucor*, *Ustilago*, *Protostropharia*, *Cephaliophora*, and *Xeromyces*) obviously decreased during diarrhea (Figure 6). Among them, two fungal genera (*Itersonilia* and *Ustilago*) even cannot be found in the gut fungal communities of diarrheic giraffes. LEfSe was applied to generate a cladogram to further investigate the variation in the fungal taxa composition (Figures 5C,D). Besides the above-mentioned significantly different funguses, we also observed that several funguses such as *Wallemia* and *Cladorrhinum* were the most dominant microbiota in the feces of patients in the DG group, whereas *Botryotrichum*, *Thelebolus*, and *Trichosporon* were significantly overrepresented in the CG group.

### Correlation Network Analysis

**FIGURE 7:**
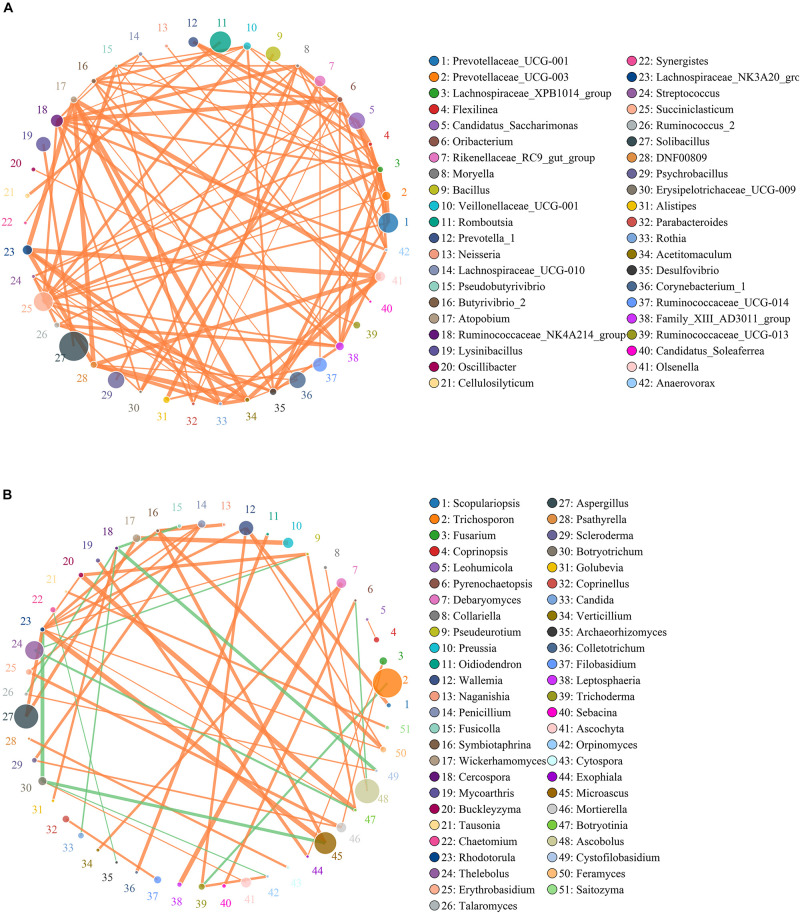
Correlation network reveals the correlation among the different bacterial **(A)** and fungal **(B)** genera. The circles with different colors indicate the name of bacterial and fungal genus, and their sizes represent relative abundance. The strength of the correlation between both genera is positively related to the thickness of the line. The green line between both genera represents a positive correlation, whereas the orange line indicates a negative correlation.

Network analysis was conducted utilizing Python to illuminate linkages among different bacterial and fungal genera in gut microbiota (Figure 7). The bacterial network consisted of 80 nodes and 1,608 edges, whereas the fungal network was composed of 80 nodes and 266 edges. Results revealed that *Ruminococcaceae_UCG-014* was positively associated with *Rikenellaceae_RC9_gut_group* (0.9231) and *Alistipes* (0.9371). *Rikenellaceae_RC9_gut_group* was positively associated with *Alistipes* (0.9790). *Microascus* was positively associated with *Rhodotorula* (0.8774). *Aspergillus* was positively associated with *Rhodotorula* (0.8194).

## Discussion

Accumulating evidence demonstrated that the stable gut microbiota was a vital barrier for host against the invasion and colonization of foreign pathogens, whereas gut microbial alternations may be the driving or central factor of multiple diseases (Dumas et al., 2006; Wang et al., 2019b; Xiang et al., 2020). Thus, investigating the gut microbial composition and diversity may contribute to expand our understanding of disease etiology and provide convenient method to evaluate the health status of host. Gut microbiota is intimately involved in several other important activities including maintaining the immunity, metabolism, and host health, which in turn depends on normal intestinal morphology (Li et al., 2021a). Diarrhea is one of the most common diseases of animals regardless of age and species, posing a great threat to public health, animal welfare, and animal husbandry. Moreover, the occurrence of diarrhea is inevitably related to gastrointestinal damage, indicating that the gut microbiota may be also altered. To date, research into the gut microbial composition and distribution in different health status has covered many species including goat, piglet, yak, and mice and demonstrated the significant variabilities of microbial community structure. However, studies regarding diarrheic influence on gut microbiota in giraffe have been insufficient to date. Taking advantage of this gap, we first characterized the variability of gut microbiota in diarrheic giraffes.

Generally, gut microbiota is dynamically diverse within limits and influenced by multiple factors such as species, age, sex, diet, and health status (Manichanh et al., 2012; Wang et al., 2020; Xiong et al., 2020; Zhu et al., 2020). Several studies have indicated that diarrhea was able to cause a significant decrease in the diversity of gut bacterial community as well as shifts in intestinal functions (Barash et al., 2017; He et al., 2020). Ma et al. (2020) revealed that the diversity of gut bacterial community in diarrheic rats was significantly decreased. Additionally, He et al. (2020) demonstrated a decreased alpha diversity of the gut bacterial community in diarrheic piglets. Consequently, diarrheic giraffes may be accompanied by significant changes in the gut microbial composition and structure. Considering the particularity of the species and the availability of samples, we selected fecal samples as the research object to assess the gut microbial composition and diversity. Consistent with previous studies, this study demonstrated a dramatically declined ACE index in the gut bacterial community of giraffe during diarrhea, implying the disorder of gut bacterial community. Several previous studies have demonstrate that the abundance and diversity of gut microbiota was positively related to intestinal functions, thereby the intestine with higher microbial diversity and abundance favors the energy utilization and performing complicated physiological functions (Barash et al., 2017; He et al., 2020). Furthermore, imbalanced gut microbiota may result in increased intestinal permeability and decreased immunity, which may further promote the invasion by members of pathogenic bacteria and conditioned pathogen (Barash et al., 2017). Therefore, diarrheic giraffe may also be at risk of intestinal dysfunction and other complications under situation of decreased gut bacterial diversity. Gut fungi, as a key component of the microbial community, was also considered to be an important contributor to intestinal health and function (Barash et al., 2017). Previous research has indicated that the gut fungal diversity of patients with diarrhea-predominant irritable bowel syndrome was significantly different from that of healthy population (Shukla et al., 2015; Hong et al., 2020). Li et al. (2018) indicated that there was no significant difference in the diversity of the gut fungal community between diarrheic and healthy yaks. Moreover, Sangster et al. (2016) observed that the gut fungal community of diarrheic patients induced by *Clostridium difficile* infection did not change significantly as compared with healthy population. In this study, we also observed that the differences of gut fungal diversity between diarrheic and healthy giraffes were not significant. Therefore, we speculated that gut bacterial community played a major role in the occurrence of giraffe diarrhea, whereas gut fungi community was secondary. Moreover, PCoA also was performed to evaluate the differences in the main components of the gut bacterial and fungal communities between both groups. Results indicated that the individuals of control group were clustered together and separated from the diarrhea group, implying a distinct difference in the primary composition of the gut bacterial and fungal communities between both groups. This study indicated that despite of shared environment and diets, the giraffes showed significant alterations in the gut bacterial and fungal communities during diarrhea. Therefore, we speculated that diarrhea was the primary driving force for changes in gut bacterial and fungal communities of giraffes.

This study demonstrated that *Firmicutes*, *Proteobacteria*, and *Bacteroidetes* were the most dominant bacterial phyla, whereas *Ascomycota* and *Basidiomycota* were the most preponderant fungal phyla in gut microbial community of giraffes, regardless of health status. These bacterial and fungal phyla were also found to be abundantly presented in the gut microbiota of cattle, goat, and yak, which was shown to be the major characteristic of the gut microbial community in ruminants (Li et al., 2018, 2021b). We further investigated the gut microbial variabilities of this common diarrhea of giraffe. The differences of specific bacteria and fungi can intuitively indicate the potential relationship between gut microbiota and diarrhea. Our results revealed distinct increases in the relative abundances of eight bacterial phyla (*Synergistetes*, *Fibrobacteres*, *Patescibacteria*, *Chloroflexi*, *Kiritimatiellaeota*, *Actinobacteria*, *Bacteroidetes*, and *Verrucomicrobia*) and significant declines in the relative abundances of two phyla (*Spirochaetes* and *Firmicutes*) during diarrhea. *Firmicutes*, consisting of a large amount of gram-positive bacteria, are responsible for converting complicated carbohydrates into reabsorbable substrates (Li et al., 2018, 2021b). Moreover, most members of *Firmicutes* are considered beneficial bacteria, which helps in regulating systemic immune responses, maintaining intestinal environment and inhibiting opportunistic pathogens (Sun et al., 2016). *Actinobacteria* synergy with one partner or host can easily be transformed into pathogenic interactions with another (Miao and Davies, 2010). Consistent with our findings, Wang et al. (2018) also observed that the abundances of *Actinobacteria* and *Verrucomicrobia* in the gut microbiota of diarrheic goats were significantly increased. *Synergistetes* has been shown to cause periodontal disease (Vartoukian et al., 2009; McCracken and Nathalia, 2020).

Importantly, this study also found a high variation in some bacterial and fungal genera between healthy and diarrheic giraffes and this variation may play crucial roles in intestinal ecosystem and function. This study demonstrated significant increases in the relative abundances of 12 genera (*Streptococcus*, *Moryella*, *Veillonellaceae_UCG-001*, *Klebsiella*, *Enterococcus*, *Catenibacterium*, *Atopobium*, *Comamonas*, *Rothia*, *Corynebacterium_1*, *Oscillibacter*, and *Terrisporobacter*) as well as significant declines in the relative abundances of eight genera (*Prevotellaceae_UCG-004*, *Rikenellaceae_RC9_gut_group*, *Coprococcus_3*, *Cellulosilyticum*, *Ruminococcaceae_UCG-014*, *Romboutsia*, *Ruminiclostridium_5*, and *Alloprevotella*) with the effect of diarrhea. *Prevotellaceae* and *Cellulosilyticum* have been reported to be involved in the degradation and digestion of carbohydrate, pectin, and cellulose (Cai et al., 2010; De Filippo et al., 2010; O’Keefe et al., 2015). *Rikenellaceae* can degrade plant-derived polysaccharides and limit the development of colitis *via* stimulating T-regulatory cell differentiation (He et al., 2015; Peng et al., 2015; Dubin et al., 2016). *Ruminococcaceae* has long been regarded as a potential beneficial bacterium due to the positive regulation of the immune system and intestinal environment (Shang et al., 2016). Furthermore, *Ruminococcaceae* has been reported to be negatively associated with liver cirrhosis, non-alcoholic fatty liver, and increased intestinal permeability (Huang et al., 2015; Shang et al., 2016). *Romboutsia*, an obligate anaerobe, contains multiple metabolic capabilities associated with carbohydrate utilization and fermentation of single amino acids (Ricaboni et al., 2016; Xin et al., 2019). *Alloprevotella* can produce moderate amounts of acetate and succinate and decrease lifetime cardiovascular disease risk (Downes et al., 2013; Ricaboni et al., 2016; Xin et al., 2019). Some genera such as *Coprococcus* and *Ruminiclostridium* have been demonstrated to produce short-chain fatty acids, which is beneficial to regulate energy intake and maintain the morphology and function of intestine and intestinal epithelial cells (Tan et al., 2014; Ye et al., 2021). Moreover, *Ruminiclostridium*, as an intestinal beneficial bacterium, is involved in the positive regulation of the growth performance and decrease of gastrointestinal diseases of animals (Tan et al., 2014). These produced metabolites play an important role in improving intestinal environment and maintaining intestinal health. Given this phenomenon, we speculated that those genera seem to participate as key factors in maintaining the balance of gut microbiota and modulating intestinal physiological activities to further prevent diarrhea. The higher abundances of *Streptococcus*, *Moryella*, *Klebsiella*, *Comamonas*, *Oscillibacter*, and *Rothia* in the gut microbiota were closely related to many diseases including septicemia, bacteremia, endocarditis, pneumonia, and cellulitis (Boudewijns et al., 2003; Fendukly et al., 2003; Broutin et al., 2020; Kjaer et al., 2020; Liu X. J. et al., 2020; Palacio et al., 2020). *Veillonellaceae* may promote the development of inflammation and its abundance increase significantly in patients with inflammatory bowel disease and irritable bowel syndrome (Boudewijns et al., 2003; Fendukly et al., 2003; Broutin et al., 2020; Kjaer et al., 2020; Liu Z. et al., 2020; Palacio et al., 2020). *Klebsiella*, a gram-negative pathogenic bacterium, mainly distributed in the respiratory tract and intestine, which may cause pneumonia, hysteritis, mastitis, and other suppurative inflammation (Nordmann et al., 2009; Qin et al., 2020). *Enterococcus* has been shown to result in life-threatening meningitis, endocarditis, and sepsis (Mohanty et al., 2005). Moreover, many antibiotics frequently used in the clinic failed treating *Enterococcus* infection because of the inherent and acquired drug resistance (Arias and Murray, 2012). *Catenibacterium* was closely related to morbid obesity and metabolic syndrome (Perler et al., 2021). The relative abundance of *Atopobium* was significantly increased in the patients with esophageal cancer (Deng et al., 2021). *Corynebacterium* can result in lung abscess and caseous lymphadenitis, which was widespread in small ruminant populations (Samies et al., 1986; Dorella et al., 2006). *Terrisporobacter*, an emerging anaerobic pathogen, can cause surgical site infection (Cheng et al., 2016).

Gut fungal community also plays important roles in intestinal function and host health, which are in line with gut bacterial community (Paterson et al., 2017). Interestingly, although the differences in gut fungal diversity between both groups were not significant, the proportion of some intestinal fungus was altered. We observed that the diarrheic giraffes displayed increased *Microascus*, *Aspergillus*, *Rhodotorula*, *Scopulariopsis*, and *Roussoella* and decreased *Itersonilia*, *Neoascochyta*, *Mucor*, *Ustilago*, *Protostropharia*, *Cephaliophora*, and *Xeromyces* as compared with healthy populations. *Microascus* has been demonstrated to cause life-threatening brain abscess and pneumonia (Baddley et al., 2000; Mohammedi et al., 2004; Ustun et al., 2006; Paterson et al., 2017). *Aspergillus* was closely related to multiple respiratory disease, while *Rhodotorula* can cause fungemia (Zaas et al., 2003; Viegas et al., 2021). *Scopulariopsis* is characterized by its inherent resistance to the available antifungal drugs and could result in pulmonary and disseminated infections (Zaas et al., 2003; Kammoun et al., 2018; Viegas et al., 2021). *Roussoella* is a novel opportunistic pathogen, which can cause subcutaneous mycoses (Ahmed et al., 2014; Vasant et al., 2017). Diarrheic giraffes with altered gut microbiota may also be accompanied by weakened immunity and disease resistance. Consequently, some opportunistic pathogens may also display pathogenicity, which may worsen the condition or cause other diseases.

Microbes inhabiting in the gastrointestinal tract such as bacteria and fungus can form a stabilized ecosystem that plays vital roles in disease prevention, pathogenic growth inhibition, and gastrointestinal homeostasis (Ahmed et al., 2014; Hu et al., 2017; Vasant et al., 2017). Generally, the intestinal bacteria and fungi can interact in a commensal, symbiotic, or antagonistic relationship, causing a stabilized ecosystem (Aziz et al., 2013; Dabke et al., 2019). The damaged stable state of gut microbial community was regarded as the pathological mediators of multiple diseases (Sircana et al., 2018). Therefore, the altered intestinal bacteria and fungi may affect other bacterial and fungal functions, which further affect the overall intestinal functions and aggravate the gut microbial alternations (Lozupone et al., 2012). We observed that the *Ruminococcaceae_UCG-014*, *Rikenellaceae_RC9_gut_group*, and *Alistipes* were dramatically affected by diarrhea, and there is a significant positive correlation among them, which implied that their functions could be further affected. In the fungal community, some pathogenic bacteria such as *Microascus*, *Aspergillus*, and *Rhodotorula* were also significantly affected by diarrhea, and those fungi also displayed a strong positive correlation. Therefore, these pathogenic bacteria may interact with each other, which further increase their pathogenicity. This study conveyed a crucial message that diarrhea not only directly changed the gut microbial diversity and abundance but also indirectly affected other functional bacteria and fungus, which may affect intestinal functions.

## Conclusion

In summary, this study characterized the dynamic alternations of gut bacterial and fungal communities during diarrhea in giraffes. Results demonstrated that the gut bacterial community in diarrheal giraffe undergoes significant changes, characterized by a decreased gut bacterial diversity and altered gut bacterial composition. Moreover, although diarrhea did not change the gut fungal diversity of giraffe, the types and proportions of some fungus have changed significantly. These results also enriched the knowledge of the gut bacterial and fungal in giraffe and convey an important message that the altered gut bacterial and fungal community may be one of the causes for the occurrence or aggravation of diarrhea. This study also provides a theoretical basis for alleviating diarrhea from the gut microbial perspective.

## Data Availability Statement

The datasets presented in this study can be found in online repositories. The names of the repository/repositories and accession number(s) can be found below: https://www.ncbi.nlm.nih.gov/, PRJNA727450.

## Ethics Statement

The animal study was reviewed and approved by the Animal Welfare and Ethics Committee of Hubei Three Gorges Polytechnic.

## Author Contributions

XJ and AL provided the research idea. YW, BL, YF, YH, HL, FL, HZ, and LW contributed to reagents, materials, and analysis tools. AL wrote the manuscript. MF-e-AK and AL revised the manuscript. All authors participated in writing and reviewing the manuscript.

## Conflict of Interest

The authors declare that the research was conducted in the absence of any commercial or financial relationships that could be construed as a potential conflict of interest.

## Publisher’s Note

All claims expressed in this article are solely those of the authors and do not necessarily represent those of their affiliated organizations, or those of the publisher, the editors and the reviewers. Any product that may be evaluated in this article, or claim that may be made by its manufacturer, is not guaranteed or endorsed by the publisher.

## References

[B1] Ahmed S. A. Stevens D. A. van de Sande W. W. Meis J. F. de Hoog G. S. (2014). Roussoella percutanea, a novel opportunistic pathogen causing subcutaneous mycoses. *Med. Mycol.* 52 689–698. 10.1093/mmy/myu035 24969729

[B2] AlZahal O. Valdes E. V. McBride B. W. (2016). Analysis of the distal gut bacterial community by 454-pyrosequencing in captive giraffes (*Giraffa camelopardalis*). *Zoo Biol.* 35 42–50. 10.1002/zoo.21252 26584008

[B3] Ambalam P. Raman M. Purama R. K. Doble M. (2016). Probiotics, prebiotics and colorectal cancer prevention. *Best Pract. Res. Clin. Gastroenterol.* 30 119–131. 10.1016/j.bpg.2016.02.009 27048903

[B4] Arias C. A. Murray B. E. (2012). The rise of the enterococcus: beyond vancomycin resistance. *Nat. Rev. Microbiol.* 10 266–278. 10.1038/nrmicro2761 22421879 PMC3621121

[B5] Arumugam M. Raes J. Pelletier E. Le Paslier D. Yamada T. Mende D. R. (2011). Enterotypes of the human gut microbiome. *Nature* 473 174–180. 10.1038/nature09944 21508958 PMC3728647

[B6] Aziz Q. Dore J. Emmanuel A. Guarner F. Quigley E. M. (2013). Gut microbiota and gastrointestinal health: current concepts and future directions. *Neurogastroenterol. Motil.* 25 4–15. 10.1111/nmo.12046 23279728

[B7] Backhed F. Roswall J. Peng Y. Feng Q. Jia H. Kovatcheva-Datchary P. (2015). Dynamics and stabilization of the human gut microbiome during the first year of life. *Cell Host Microbe* 17 690–703. 10.1016/j.chom.2015.04.004 25974306

[B8] Baddley J. W. Moser S. A. Sutton D. A. Pappas P. G. (2000). Microascus cinereus (*Anamorph scopulariopsis*) brain abscess in a bone marrow transplant recipient. *J. Clin. Microbiol.* 38 395–397. 10.1128/jcm.38.1.395-397.2000 10618123 PMC88731

[B9] Barash N. R. Maloney J. G. Singer S. M. Dawson S. C. (2017). Giardia alters commensal microbial diversity throughout the murine gut. *Infect. Immun.* 85:e00948–16. 10.1128/IAI.00948-16 28396324 PMC5442636

[B10] Boudewijns M. Magerman K. Verhaegen J. Debrock G. Peetermans W. E. Donkersloot P. (2003). Rothia dentocariosa, endocarditis and mycotic aneurysms: case report and review of the literature. *Clin. Microbiol. Infect.* 9 222–229. 10.1046/j.1469-0691.2003.00503.x 12667255

[B11] Broutin L. Deroche L. Michaud A. Le Moal G. Burucoa C. Gayet L. E. (2020). First description of bacteremia caused by *Oscillibacter valericigenes* in a patient hospitalized for leg amputation. *Anaerobe* 64:102244. 10.1016/j.anaerobe.2020.102244 32712374

[B12] Brown D. M. Brenneman R. A. Koepfli K. P. Pollinger J. P. Mila B. Georgiadis N. J. (2007). Extensive population genetic structure in the giraffe. *BMC Biol.* 5:57. 10.1186/1741-7007-5-57 18154651 PMC2254591

[B13] Bu D. Zhang X. Ma L. Park T. Wang L. Wang M. (2020). Repeated inoculation of young calves with rumen microbiota does not significantly modulate the rumen prokaryotic microbiota consistently but decreases diarrhea. *Front. Microbiol.* 11:1403. 10.3389/fmicb.2020.01403 32670244 PMC7326819

[B14] Cai S. Li J. Hu F. Z. Zhang K. Luo Y. Janto B. (2010). Cellulosilyticum ruminicola, a newly described rumen bacterium that possesses redundant fibrolytic-protein-encoding genes and degrades lignocellulose with multiple carbohydrate- borne fibrolytic enzymes. *Appl. Environ. Microbiol.* 76 3818–3824. 10.1128/AEM.03124-09 20400560 PMC2893506

[B15] Cheng M. P. Domingo M. C. Levesque S. Yansouni C. P. (2016). A case report of a deep surgical site infection with *Terrisporobacter glycolicus*/*T. Mayombei* and review of the literature. *BMC Infect. Dis* 16:529. 10.1186/s12879-016-1865-8 27686579 PMC5043541

[B16] Cryan J. F. Dinan T. G. (2012). Mind-altering microorganisms: the impact of the gut microbiota on brain and behaviour. *Nat. Rev. Neurosci.* 13 701–712. 10.1038/nrn3346 22968153

[B17] Dabke K. Hendrick G. Devkota S. (2019). The gut microbiome and metabolic syndrome. *J. Clin. Invest.* 129 4050–4057. 10.1172/JCI129194 31573550 PMC6763239

[B18] De Filippo C. Cavalieri D. Di Paola M. Ramazzotti M. Poullet J. B. Massart S. (2010). Impact of diet in shaping gut microbiota revealed by a comparative study in children from Europe and rural Africa. *Proc. Natl. Acad. Sci. U.S.A.* 107 14691–14696. 10.1073/pnas.1005963107 20679230 PMC2930426

[B19] Delzenne N. M. Neyrinck A. M. Cani P. D. (2011). Modulation of the gut microbiota by nutrients with prebiotic properties: consequences for host health in the context of obesity and metabolic syndrome. *Microb. Cell Fact.* 10 Suppl 1:S10. 10.1186/1475-2859-10-S1-S10 21995448 PMC3231917

[B20] Deng Y. Tang D. Hou P. Shen W. Li H. Wang T. (2021). Dysbiosis of gut microbiota in patients with esophageal cancer. *Microb. Pathog.* 150:104709. 10.1016/j.micpath.2020.104709 33378710

[B21] Dorella F. A. Pacheco L. G. Oliveira S. C. Miyoshi A. Azevedo V. (2006). Corynebacterium pseudotuberculosis: Microbiology, biochemical properties, pathogenesis and molecular studies of virulence. *Vet. Res.* 37 201–218. 10.1051/vetres:2005056 16472520

[B22] Downes J. Dewhirst F. E. Tanner A. Wade W. G. (2013). Description of *Alloprevotella rava* gen. Nov., Sp. Nov., Isolated from the human oral cavity, and reclassification of *Prevotella tannerae* Moore et al. 1994 as *Alloprevotella tannerae* gen. Nov., Comb. Nov. *Int. J. Syst. Evol. Microbiol.* 63 1214–1218. 10.1099/ijs.0.041376-0 22753527 PMC3709537

[B23] Dubin K. Callahan M. K. Ren B. Khanin R. Viale A. Ling L. (2016). Intestinal microbiome analyses identify melanoma patients at risk for checkpoint-blockade-induced colitis. *Nat. Commun.* 7:10391. 10.1038/ncomms10391 26837003 PMC4740747

[B24] Dumas M. E. Barton R. H. Toye A. Cloarec O. Blancher C. Rothwell A. (2006). Metabolic profiling reveals a contribution of gut microbiota to fatty liver phenotype in insulin-resistant mice. *Proc. Natl. Acad. Sci. U.S.A.* 103 12511–12516. 10.1073/pnas.0601056103 16895997 PMC1567909

[B25] Fendukly F. Karlsson I. Hanson H. S. Kronvall G. Dornbusch K. (2003). Patterns of mutations in target genes in septicemia isolates of *Escherichia coli* and *Klebsiella pneumoniae* with resistance or reduced susceptibility to ciprofloxacin. *Apmis* 111 857–866. 10.1034/j.1600-0463.2003.1110904.x 14510643

[B26] Gallardo P. Izquierdo M. Vidal R. M. Soto F. Ossa J. C. Farfan M. J. (2020). Gut Microbiota-Metabolome changes in children with diarrhea by diarrheagenic *E. coli*. *Front. Cell Infect. Microbiol.* 10: 485. 10.3389/fcimb.2020.00485 33072619 PMC7531578

[B27] Garrett W. S. Gordon J. I. Glimcher L. H. (2010). Homeostasis and inflammation in the intestine. *Cell* 140 859–870. 10.1016/j.cell.2010.01.023 20303876 PMC2845719

[B28] Guo W. Li Y. Wang L. Wang J. Xu Q. Yan T. (2015). Evaluation of composition and individual variability of rumen microbiota in yaks by 16S rRNA high-throughput sequencing technology. *Anaerobe* 34 74–79. 10.1016/j.anaerobe.2015.04.010 25911445

[B29] Han Z. Li K. Shahzad M. Zhang H. Luo H. Qiu G. (2017). Analysis of the intestinal microbial community in healthy and diarrheal perinatal yaks by high-throughput sequencing. *Microb. Pathog.* 111 60–70. 10.1016/j.micpath.2017.08.025 28823792

[B30] He B. Nohara K. Ajami N. J. Michalek R. D. Tian X. Wong M. (2015). Transmissible microbial and metabolomic remodeling by soluble dietary fiber improves metabolic homeostasis. *Sci. Rep.* 5:10604. 10.1038/srep10604 26040234 PMC4455235

[B31] He K. Yan W. Sun C. Liu J. Bai R. Wang T. (2020). Alterations in the diversity and composition of gut microbiota in weaned piglets infected with *Balantioides coli*. *Vet. Parasitol.* 288:109298. 10.1016/j.vetpar.2020.109298 33171414

[B32] Hong G. Li Y. Yang M. Li G. Qian W. Xiong H. (2020). Gut fungal dysbiosis and altered bacterial-fungal interaction in patients with diarrhea-predominant irritable bowel syndrome: an explorative study. *Neurogastroenterol. Motil.* 32:e13891. 10.1111/nmo.13891 32449259

[B33] Hu L. Geng S. Li Y. Cheng S. Fu X. Yue X. (2017). Exogenous fecal microbiota transplantation from local adult pigs to crossbred newborn piglets. *Front. Microbiol.* 8:2663. 10.3389/fmicb.2017.02663 29375527 PMC5767267

[B34] Huang C. Song P. Fan P. Hou C. Thacker P. Ma X. (2015). Dietary sodium butyrate decreases postweaning diarrhea by modulating intestinal permeability and changing the bacterial communities in weaned piglets. *J. Nutr.* 145 2774–2780. 10.3945/jn.115.217406 26491121

[B35] Kammoun S. Rekik M. Trabelsi H. Neji S. Feki J. Ayadi A. (2018). Orbital cellulitis secondary to a fungal sinusitis caused by *Scopulariopsis*: the first case in Tunisia. *J. Mycol. Med.* 28 384–386. 10.1016/j.mycmed.2018.04.006 29709267

[B36] Kjaer H. S. Lofberg S. V. Nielsen D. K. Kobbero H. Justesen U. S. (2020). Bacteraemia with moryella indoligenes and *Fastidiosipila sanguinis*: a case report. *Access Microbiol.* 2:acmi000108. 10.1099/acmi.0.000108 32974574 PMC7494188

[B37] Li A. Wang Y. He Y. Liu B. Iqbal M. Mehmood K. (2021a). Environmental fluoride exposure disrupts the intestinal structure and gut microbial composition in ducks. *Chemosphere* 277:130222. 10.1016/j.chemosphere.2021.130222 33794430

[B38] Li A. Yang Y. Zhang Y. Lv S. Jin T. Li K. (2021b). Microbiome analysis reveals the alterations in gut microbiota in different intestinal segments of Yimeng black goats. *Microb. Pathog.* 155:104900. 10.1016/j.micpath.2021.104900 33894292

[B39] Li K. Mehmood K. Zhang H. Jiang X. Shahzad M. Dong X. (2018). Characterization of fungus microbial diversity in healthy and diarrheal yaks in Gannan region of Tibet Autonomous Prefecture. *Acta Trop.* 182 14–26. 10.1016/j.actatropica.2018.02.017 29454733

[B40] Liu X. J. Qiao X. W. Huang T. M. Li L. Jiang S. P. (2020). Comamonas kerstersii bacteremia. *Med. Mal. Infect.* 50 288–290. 10.1016/j.medmal.2019.12.005 32169298

[B41] Liu Z. Li A. Wang Y. Iqbal M. Zheng A. Zhao M. (2020). Comparative analysis of microbial community structure between healthy and Aeromonas veronii-infected Yangtze finless porpoise. *Microb. Cell Fact.* 19:123. 10.1186/s12934-020-01383-4 32503532 PMC7275351

[B42] Lozupone C. A. Stombaugh J. I. Gordon J. I. Jansson J. K. Knight R. (2012). Diversity, stability and resilience of the human gut microbiota. *Nature* 489 220–230. 10.1038/nature11550 22972295 PMC3577372

[B43] Ma Z. Wang H.-J. Ma X.-J. Li Y. Yang H.-J. Li H. (2020). Modulation of gut microbiota and intestinal barrier function during alleviation of antibiotic-associated diarrhea with Rhizoma Zingiber officinale (Ginger) extract. *Food Funct.* 11, 10839–10851. 10.1039/d0fo01536a 33241234

[B44] Manichanh C. Borruel N. Casellas F. Guarner F. (2012). The gut microbiota in IBD. *Nat. Rev. Gastroenterol. Hepatol.* 9 599–608. 10.1038/nrgastro.2012.152 22907164

[B45] McCracken B. A. Nathalia G. M. (2020). Phylum Synergistetes in the oral cavity: a possible contributor to periodontal disease. *Anaerobe* 68:102250. 10.1016/j.anaerobe.2020.102250 32791127

[B46] Miao V. Davies J. (2010). Actinobacteria: the good, the bad, and the ugly. *Antonie Van Leeuwenhoek* 98 143–150. 10.1007/s10482-010-9440-6 20390355

[B47] Mohammedi I. Piens M. A. Audigier-Valette C. Gantier J. C. Argaud L. Martin O. (2004). Fatal Microascus trigonosporus (anamorph Scopulariopsis) pneumonia in a bone marrow transplant recipient. *Eur. J. Clin. Microbiol. Infect. Dis.* 23 215–217. 10.1007/s10096-003-1096-y 14986165

[B48] Mohanty S. Dhawan B. Kapil A. Das B. K. Pandey P. Gupta A. (2005). Brain abscess due to *Enterococcus avium*. *Am. J. Med. Sci.* 329 161–162. 10.1097/00000441-200503000-00011 15767825

[B49] Mulherin E. Bryan J. Beltman M. O’Grady L. Pidgeon E. Garon L. (2008). Molecular characterisation of a bovine-like rotavirus detected from a giraffe. *BMC Vet. Res.* 4:46. 10.1186/1746-6148-4-46 19014526 PMC2632637

[B50] Musso G. Gambino R. Cassader M. (2011). Interactions between gut microbiota and host metabolism predisposing to obesity and diabetes. *Annu. Rev. Med.* 62 361–380. 10.1146/annurev-med-012510-175505 21226616

[B51] Nordmann P. Cuzon G. Naas T. (2009). The real threat of *Klebsiella pneumoniae* carbapenemase-producing bacteria. *Lancet Infect. Dis.* 9 228–236. 10.1016/S1473-3099(09)70054-4 19324295

[B52] O’Keefe S. J. Li J. V. Lahti L. Ou J. Carbonero F. Mohammed K. (2015). Fat, fibre and cancer risk in african americans and rural africans. *Nat. Commun.* 6:6342. 10.1038/ncomms7342 25919227 PMC4415091

[B53] Palacio R. Cabezas L. Cornejo C. Seija V. (2020). [Comamonas kerstersii bacteremia in a young man with acute appendicitis]. *Rev. Chilena. Infectol.* 37 182–185. 10.4067/s0716-10182020000200182 32730487

[B54] Paterson M. J. Oh S. Underhill D. M. (2017). Host-microbe interactions: commensal fungi in the gut. *Curr. Opin. Microbiol.* 40 131–137. 10.1016/j.mib.2017.11.012 29175338 PMC5733715

[B55] Peng B. Huang S. Liu T. Geng A. (2015). Bacterial xylose isomerases from the mammal gut *Bacteroidetes cluster* function in *Saccharomyces cerevisiae* for effective xylose fermentation. *Microb. Cell Fact.* 14:70. 10.1186/s12934-015-0253-1 25981595 PMC4436767

[B56] Perler B. K. Reinhart E. M. Montgomery M. Maynard M. Shapiro J. M. Belenky P. (2021). Evaluation of the microbiome in men taking pre-exposure prophylaxis for HIV prevention. *AIDS Behav.* 25 2005–2013. 10.1007/s10461-020-03130-7 33394167 PMC8169604

[B57] Qin X. Wu S. Hao M. Zhu J. Ding B. Yang Y. (2020). The colonization of carbapenem-resistant *klebsiella pneumoniae*: epidemiology, resistance mechanisms, and risk factors in patients admitted to intensive care units in china. *J. Infect. Dis.* 221 S206–S214. 10.1093/infdis/jiz622 32176790

[B58] Ricaboni D. Mailhe M. Khelaifia S. Raoult D. Million M. (2016). Romboutsia timonensis, a new species isolated from human gut. *New Microbes New Infect.* 12 6–7. 10.1016/j.nmni.2016.04.001 27200178 PMC4864248

[B59] Samies J. H. Hathaway B. N. Echols R. M. Veazey J. J. Pilon V. A. (1986). Lung abscess due to Corynebacterium equi. Report of the first case in a patient with acquired immune deficiency syndrome. *Am. J. Med.* 80 685–688. 10.1016/0002-9343(86)90825-9 3963045

[B60] Sangster W. Hegarty J. P. Schieffer K. M. Wright J. R. Hackman J. Toole D. R. (2016). Bacterial and fungal microbiota changes distinguish C. Difficile infection from other forms of diarrhea: results of a prospective inpatient study. *Front. Microbiol.* 7:789. 10.3389/fmicb.2016.00789 27252696 PMC4879479

[B61] Shang Q. Shan X. Cai C. Hao J. Li G. Yu G. (2016). Dietary fucoidan modulates the gut microbiota in mice by increasing the abundance of *Lactobacillus* and *Ruminococcaceae*. *Food Funct.* 7 3224–3232. 10.1039/c6fo00309e 27334000

[B62] Shao H. Zhang C. Xiao N. Tan Z. (2020). Gut microbiota characteristics in mice with antibiotic-associated diarrhea. *BMC Microbiol.* 20:313. 10.1186/s12866-020-01999-x 33059603 PMC7559773

[B63] Shukla R. Ghoshal U. Dhole T. N. Ghoshal U. C. (2015). Fecal microbiota in patients with irritable bowel syndrome compared with healthy controls using Real-Time polymerase chain reaction: an evidence of dysbiosis. *Dig. Dis. Sci.* 60 2953–2962. 10.1007/s10620-015-3607-y 25784074

[B64] Sircana A. Framarin L. Leone N. Berrutti M. Castellino F. Parente R. (2018). Altered gut microbiota in type 2 diabetes: just a coincidence? *Curr. Diab. Rep.* 18:98. 10.1007/s11892-018-1057-6 30215149

[B65] Sun B. Wang X. Bernstein S. Huffman M. A. Xia D. P. Gu Z. (2016). Marked variation between winter and spring gut microbiota in free-ranging Tibetan Macaques (*Macaca thibetana*). *Sci. Rep.* 6:26035. 10.1038/srep26035 27180722 PMC4867428

[B66] Tan J. McKenzie C. Potamitis M. Thorburn A. N. Mackay C. R. Macia L. (2014). The role of short-chain fatty acids in health and disease. *Adv. Immunol.* 121 91–119. 10.1016/B978-0-12-800100-4.00003-9 24388214

[B67] Tremaroli V. Backhed F. (2012). Functional interactions between the gut microbiota and host metabolism. *Nature* 489 242–249. 10.1038/nature11552 22972297

[B68] Ustun C. Huls G. Stewart M. Marr K. A. (2006). Resistant *Microascus cirrosus* pneumonia can be treated with a combination of surgery, multiple anti-fungal agents and a growth factor. *Mycopathologia* 162 299–302. 10.1007/s11046-006-0067-0 17039277

[B69] Vartoukian S. R. Palmer R. M. Wade W. G. (2009). Diversity and morphology of members of the phylum “synergistetes” in periodontal health and disease. *Appl. Environ. Microbiol.* 75 3777–3786. 10.1128/AEM.02763-08 19346352 PMC2687275

[B70] Vasant J. A. Maggiani F. Borman A. M. (2017). Subcutaneous mycotic cyst caused by roussoella percutanea in a UK renal transplant patient. *Mycopathologia* 182 721–725. 10.1007/s11046-017-0121-0 28190203

[B71] Viegas C. Caetano L. A. Viegas S. (2021). Occupational exposure to *Aspergillus* section *Fumigati*: tackling the knowledge gap in Portugal. *Environ Res* 194 110674. 10.1016/j.envres.2020.110674 33440201

[B72] Wang J. Zhu G. Sun C. Xiong K. Yao T. Su Y. (2020). TAK-242 ameliorates DSS-induced colitis by regulating the gut microbiota and the JAK2/STAT3 signaling pathway. *Microb. Cell Fact.* 19:158. 10.1186/s12934-020-01417-x 32762699 PMC7412642

[B73] Wang Y. Li A. Liu J. Mehmood K. Wangdui B. Shi H. (2019a). *L. Pseudomesenteroides* and *L. Johnsonii* isolated from yaks in Tibet modulate gut microbiota in mice to ameliorate enteroinvasive *Escherichia coli*-induced diarrhea. *Microb. Pathog.* 132 1–9. 10.1016/j.micpath.2019.04.020 30999021

[B74] Wang Y. Li A. Zhang L. Waqas M. Mehmood K. Iqbal M. (2019b). Probiotic potential of Lactobacillus on the intestinal microflora against *Escherichia coli* induced mice model through high-throughput sequencing. *Microb. Pathog.* 137:103760. 10.1016/j.micpath.2019.103760 31562897

[B75] Wang Y. Zhang H. Zhu L. Xu Y. Liu N. Sun X. (2018). Dynamic distribution of gut microbiota in goats at different ages and health states. *Front. Microbiol.* 9:2509. 10.3389/fmicb.2018.02509 30405569 PMC6207909

[B76] Xiang L. Wu Q. Osada H. Yoshida M. Pan W. Qi J. (2020). Peanut skin extract ameliorates the symptoms of type 2 diabetes mellitus in mice by alleviating inflammation and maintaining gut microbiota homeostasis. *Aging (Albany NY)* 12 13991–14018. 10.18632/aging.103521 32699185 PMC7425515

[B77] Xin J. Chai Z. Zhang C. Zhang Q. Zhu Y. Cao H. (2019). Comparing the microbial community in four stomach of dairy cattle, yellow cattle and three yak herds in Qinghai-Tibetan plateau. *Front. Microbiol.* 10:1547. 10.3389/fmicb.2019.01547 31354656 PMC6636666

[B78] Xiong L. You J. Zhang W. Zhu Q. Blachier F. Yin Y. (2020). Intrauterine growth restriction alters growth performance, plasma hormones, and small intestinal microbial communities in growing-finishing pigs. *J. Anim. Sci. Biotechnol.* 11:86. 10.1186/s40104-020-00490-x 32832077 PMC7437023

[B79] Xue N. Y. Liu F. Tao W. F. Zhao Q. Qiu H. Y. Hu Y. (2020). Molecular detection of *Cryptosporidium* spp. And *Enterocytozoon bieneusi* in longjiang wagyu cattle in Northeastern China. *Microb. Pathog.* 149:104526. 10.1016/j.micpath.2020.104526 33010364

[B80] Yang Q. Huang X. Zhao S. Sun W. Yan Z. Wang P. (2017). Structure and function of the fecal microbiota in diarrheic neonatal piglets. *Front. Microbiol.* 8:502. 10.3389/fmicb.2017.00502 28392784 PMC5364137

[B81] Ye X. Zhou L. Zhang Y. Xue S. Gan Q. F. Fang S. (2021). Effect of host breeds on gut microbiome and serum metabolome in meat rabbits. *BMC Vet. Res.* 17:24. 10.1186/s12917-020-02732-6 33413361 PMC7791989

[B82] Zaas A. K. Boyce M. Schell W. Lodge B. A. Miller J. L. Perfect J. R. (2003). Risk of fungemia due to Rhodotorula and antifungal susceptibility testing of Rhodotorula isolates. *J. Clin. Microbiol.* 41 5233–5235. 10.1128/jcm.41.11.5233-5235.2003 14605170 PMC262498

[B83] Zhang L. Jiang X. Li A. Waqas M. Gao X. Li K. (2020). Characterization of the microbial community structure in intestinal segments of yak (*Bos grunniens*). *Anaerobe* 61:102115. 10.1016/j.anaerobe.2019.102115 31711887

[B84] Zhu L. Xu F. Wan W. Yu B. Tang L. Yang Y. (2020). Gut microbial characteristics of adult patients with allergy rhinitis. *Microb. Cell Fact.* 19:171. 10.1186/s12934-020-01430-0 32873292 PMC7466420

